# Tetra­aqua­tetra­kis­(4,4′-bipyridine dioxide-κ*O*)terbium(III) octa­cyanido­molybdate(V)

**DOI:** 10.1107/S1600536811020022

**Published:** 2011-06-04

**Authors:** Su-Yan Qian, Ai-Hua Yuan

**Affiliations:** a School of Biological and Chemical Engineering, Jiangsu University of Science and Technology, Zhenjiang 212003, People’s Republic of China

## Abstract

In the title compound, [Tb(C_10_H_8_N_2_O_2_)_4_(H_2_O)_4_][Mo(CN)_8_], both metal atoms are eight-coordinated. The Tb^III^ atom displays a dodecahedral geometry, while the Mo^V^ ion exhibits a distorted square-anti­prismatic geometry. The Tb atoms are located on a special position of site symmetry 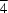, whereas the Mo atoms are located on a twofold rotation axis. The cations are linked by O—H⋯O hydrogen bonds.

## Related literature

For general background to octa­cyanidometallate-based complexes involving lanthanide ions, see: Chelebaeva *et al.* (2009 ▶); Ma *et al.* (2009 ▶); Qian *et al.* (2010 ▶); Wang *et al.* (2006 ▶); Zhou *et al.* (2010 ▶). For the preparation of the title compound, see: Bok *et al.* (1975 ▶). For related structures, see: Kozieł *et al.* (2010 ▶); Przychodzeń *et al.* (2007 ▶).
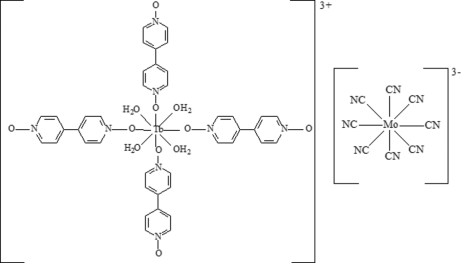


## Experimental

### 

#### Crystal data

[Tb(C_10_H_8_N_2_O_2_)_4_(H_2_O)_4_][Mo(CN)_8_]
*M* *_r_* = 1287.72
Tetragonal, 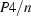

*a* = 17.9226 (7) Å
*c* = 7.8877 (6) Å
*V* = 2533.7 (2) Å^3^
*Z* = 2
Mo *K*α radiation
μ = 1.71 mm^−1^
*T* = 291 K
0.22 × 0.21 × 0.12 mm

#### Data collection

Bruker SMART APEX CCD diffractometer
Absorption correction: multi-scan (*SADABS*; Bruker, 2004 ▶) *T*_min_ = 0.693, *T*_max_ = 0.843
21243 measured reflections
2921 independent reflections
2730 reflections with *I* > 2σ(*I*)
*R*_int_ = 0.026

#### Refinement

*R*[*F* ^2^ > 2σ(*F* ^2^)] = 0.016
*wR*(*F* ^2^) = 0.043
*S* = 1.08
2921 reflections
177 parameters
H-atom parameters constrained
Δρ_max_ = 0.36 e Å^−3^
Δρ_min_ = −0.29 e Å^−3^

### 

Data collection: *SMART* (Bruker, 2004 ▶); cell refinement: *SAINT* (Bruker, 2004 ▶); data reduction: *SAINT*; program(s) used to solve structure: *SHELXS97* (Sheldrick, 2008 ▶); program(s) used to refine structure: *SHELXL97* (Sheldrick, 2008 ▶); molecular graphics: *DIAMOND* (Brandenburg, 2006 ▶); software used to prepare material for publication: *SHELXTL* (Sheldrick, 2008 ▶).

## Supplementary Material

Crystal structure: contains datablock(s) I, global. DOI: 10.1107/S1600536811020022/bt5505sup1.cif

Structure factors: contains datablock(s) I. DOI: 10.1107/S1600536811020022/bt5505Isup2.hkl

Additional supplementary materials:  crystallographic information; 3D view; checkCIF report

## Figures and Tables

**Table 1:** Hydrogen-bond geometry (Å, °)

*D*—H⋯*A*	*D*—H	H⋯*A*	*D*⋯*A*	*D*—H⋯*A*
O3—H3*WA*⋯O2^i^	0.83	1.85	2.6702 (15)	169
O3—H3*WB*⋯O2^ii^	0.84	1.92	2.7417 (16)	164

Symmetry codes: (i) 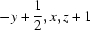; (ii) 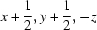.

**Fig. 1.**
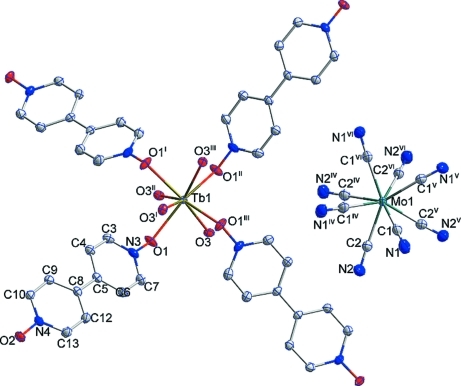
ORTEP diagram of the title compound. Hydrogen atoms have been omitted for clarity and thermal ellipsoids are presented at the 30% probability level.

**Fig. 2.**
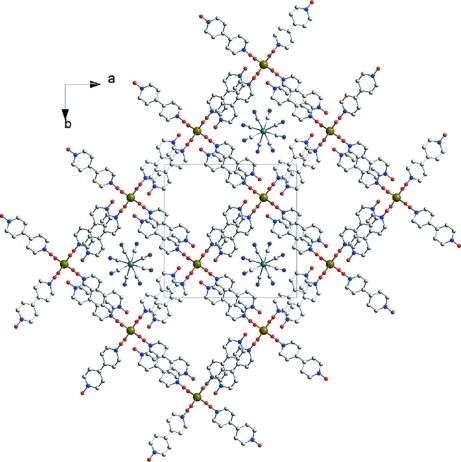
Perspective view of the title compound in ab plane. Hydrogen atoms and coordinated water molecules have been omitted for clarity.

## supplementary crystallographic information

### Comment

In the past few years, octacyanometallate-based complexes have attracted considerable attention in the field of cyanide-bridged hetero-metallic system. Many assemblies based on [*M*^V^(CN)_8_]^3-^ (*M* = Mo or W) and lanthanide ions can take a variety of  structures (Chelebaeva *et al.*, 2009; Kozieł *et al.*, 2010; Ma *et al.*, 2009; Przychodzeń *et al.*, 2007; Qian *et al.*, 2010; Wang *et al.*, 2006; Zhou *et al.*, 2010). Recently, we have used [Mo(CN)_8_]^3-^ as a building block to react with Tb^3+^ and 4,4'-bipyridine dioxide (4,4'-dpdo) obtaining a new ionic complex, [Tb(4,4'-bpdo)_4_(H_2_O)_4_][Mo(CN)_8_.

In the structure, the eight-coordinated Tb^III^ center displays a  decahedron geometry, while each [Mo^V^(CN)_8_] moiety exhibits a distorted square antiprismic geometry. The average values of Mo—C and C—N bond distances are 2.171 and 1.155 Å, respectively, while the Mo—C—N units are nearly linear. The anions are linked by O-H..O hydrogen bonds.

### Experimental

Single crystals of the title compound were prepared at room temperature in the dark by slow diffusion of a H_2_O solution (3 ml) containing Tb(NO_3_)_3_.6H_2_O (0.05 mmol) and 4,4'-dpdo (0.05 mmol) into a CH_3_CN solution (15 ml) of [HN(*n*-C_4_H_9_)_3_]_3_[Mo(CN)_8_].4H_2_O (0.05 mmol) (Bok *et al.*, 1975). After two weeks, yellow block crystals were obtained.

### Refinement

The H atoms of 4,4'-bipyridine dioxide ligands were ideally positioned   with C—H = 0.93 Å and included in the refinement using a riding model with *U*(H) set to 1.2  *U*_eq_(C). The H atoms bound to oxygen atoms were located from difference maps and refined as riding  with *U*(H) set to 1.2  *U*_eq_(O).

### Crystal data

**Table:**

[Tb(C_10_H_8_N_2_O_2_)_4_(H_2_O)_4_][Mo(CN)_8_]	*D*_x_ = 1.688 Mg m^−^^3^
*M**_r_* = 1287.72	Mo *K*α radiation, λ = 0.71073 Å
Tetragonal, *P*4/*n*	Cell parameters from 9881 reflections
Hall symbol: -P 4a	θ = 2.3–27.5°
*a* = 17.9226 (7) Å	µ = 1.71 mm^−^^1^
*c* = 7.8877 (6) Å	*T* = 291 K
*V* = 2533.7 (2) Å^3^	Block, yellow
*Z* = 2	0.22 × 0.21 × 0.12 mm
*F*(000) = 1286	

### Data collection

**Table:**

Bruker SMART APEX CCD diffractometer	2730 reflections with *I* > 2σ(*I*)
graphite	*R*_int_ = 0.026
φ and ω scans	θ_max_ = 27.5°, θ_min_ = 1.6°
Absorption correction: multi-scan (*SADABS*; Bruker, 2004)	*h* = −23→23
*T*_min_ = 0.693, *T*_max_ = 0.843	*k* = −23→23
21243 measured reflections	*l* = −10→10
2921 independent reflections	

### Refinement

**Table:**

Refinement on *F*^2^	Primary atom site location: structure-invariant direct methods
Least-squares matrix: full	Secondary atom site location: difference Fourier map
*R*[*F*^2^ > 2σ(*F*^2^)] = 0.016	Hydrogen site location: inferred from neighbouring sites
*wR*(*F*^2^) = 0.043	H-atom parameters constrained
*S* = 1.08	*w* = 1/[σ^2^(*F*_o_^2^) + (0.0183*P*)^2^ + 1.8165*P*] where *P* = (*F*_o_^2^ + 2*F*_c_^2^)/3
2921 reflections	(Δ/σ)_max_ = 0.001
177 parameters	Δρ_max_ = 0.36 e Å^−^^3^
0 restraints	Δρ_min_ = −0.29 e Å^−^^3^

### Special details

**Table:**

Geometry. All e.s.d.'s (except the e.s.d. in the dihedral angle between two l.s. planes) are estimated using the full covariance matrix. The cell e.s.d.'s are taken into account individually in the estimation of e.s.d.'s in distances, angles and torsion angles; correlations between e.s.d.'s in cell parameters are only used when they are defined by crystal symmetry. An approximate (isotropic) treatment of cell e.s.d.'s is used for estimating e.s.d.'s involving l.s. planes.
Refinement. Refinement of *F*^2^ against ALL reflections. The weighted *R*-factor *wR* and goodness of fit *S* are based on *F*^2^, conventional *R*-factors *R* are based on *F*, with *F* set to zero for negative *F*^2^. The threshold expression of *F*^2^ > σ(*F*^2^) is used only for calculating *R*-factors(gt) *etc*. and is not relevant to the choice of reflections for refinement. *R*-factors based on *F*^2^ are statistically about twice as large as those based on *F*, and *R*- factors based on ALL data will be even larger.

### Fractional atomic coordinates and isotropic or equivalent isotropic displacement parameters (Å^2^)

**Table:**

	*x*	*y*	*z*	*U*_iso_*/*U*_eq_	
Tb1	0.7500	0.2500	0.0000	0.01136 (5)	
Mo1	0.7500	0.7500	−0.24987 (4)	0.02019 (7)	
O1	0.64962 (6)	0.17736 (7)	0.10113 (14)	0.0251 (3)	
O2	0.27973 (7)	−0.11246 (7)	−0.50459 (14)	0.0223 (2)	
O3	0.70614 (6)	0.31664 (6)	0.24591 (13)	0.0183 (2)	
N1	0.60473 (10)	0.81932 (10)	−0.4592 (3)	0.0422 (4)	
N2	0.59644 (10)	0.70470 (10)	−0.0373 (3)	0.0393 (4)	
N3	0.60104 (8)	0.13848 (8)	0.00837 (16)	0.0201 (3)	
N4	0.33441 (7)	−0.07064 (7)	−0.44088 (17)	0.0176 (2)	
C1	0.65693 (10)	0.79577 (9)	−0.3922 (3)	0.0300 (4)	
C2	0.65123 (10)	0.71973 (9)	−0.1057 (3)	0.0293 (4)	
C3	0.62224 (9)	0.07458 (9)	−0.0674 (2)	0.0234 (3)	
H3	0.6709	0.0575	−0.0537	0.028*	
C4	0.57297 (9)	0.03404 (9)	−0.1648 (2)	0.0215 (3)	
H4	0.5888	−0.0098	−0.2166	0.026*	
C5	0.49930 (8)	0.05822 (8)	−0.1867 (2)	0.0175 (3)	
C6	0.47918 (9)	0.12409 (9)	−0.1031 (2)	0.0248 (3)	
H6	0.4306	0.1419	−0.1129	0.030*	
C7	0.53013 (10)	0.16301 (10)	−0.0063 (2)	0.0257 (4)	
H7	0.5155	0.2064	0.0491	0.031*	
C8	0.44383 (8)	0.01450 (8)	−0.28394 (19)	0.0168 (3)	
C9	0.45913 (8)	−0.05724 (8)	−0.3460 (2)	0.0182 (3)	
H9	0.5070	−0.0768	−0.3356	0.022*	
C10	0.40389 (8)	−0.09908 (8)	−0.4224 (2)	0.0189 (3)	
H10	0.4144	−0.1469	−0.4613	0.023*	
C12	0.37208 (9)	0.04249 (8)	−0.3126 (2)	0.0209 (3)	
H12	0.3604	0.0907	−0.2780	0.025*	
C13	0.31847 (9)	−0.00028 (9)	−0.3913 (2)	0.0217 (3)	
H13	0.2712	0.0193	−0.4103	0.026*	
H3WA	0.6727	0.3040	0.3134	0.033*	
H3WB	0.7363	0.3368	0.3136	0.033*	

### Atomic displacement parameters (Å^2^)

**Table:**

	*U*^11^	*U*^22^	*U*^33^	*U*^12^	*U*^13^	*U*^23^
Tb1	0.01183 (5)	0.01183 (5)	0.01043 (7)	0.000	0.000	0.000
Mo1	0.01407 (8)	0.01407 (8)	0.03243 (15)	0.000	0.000	0.000
O1	0.0234 (6)	0.0348 (6)	0.0171 (5)	−0.0157 (5)	−0.0019 (5)	−0.0008 (5)
O2	0.0190 (5)	0.0247 (6)	0.0231 (6)	−0.0067 (4)	−0.0023 (4)	−0.0059 (4)
O3	0.0179 (5)	0.0232 (5)	0.0139 (5)	−0.0007 (4)	0.0019 (4)	−0.0032 (4)
N1	0.0310 (9)	0.0290 (8)	0.0666 (12)	−0.0020 (7)	−0.0123 (9)	0.0126 (8)
N2	0.0297 (8)	0.0313 (8)	0.0569 (11)	0.0000 (7)	0.0099 (8)	0.0101 (8)
N3	0.0188 (6)	0.0246 (7)	0.0170 (6)	−0.0095 (5)	−0.0005 (5)	0.0018 (5)
N4	0.0183 (6)	0.0198 (6)	0.0146 (6)	−0.0043 (5)	−0.0004 (5)	−0.0006 (5)
C1	0.0243 (8)	0.0195 (8)	0.0462 (11)	−0.0031 (6)	−0.0026 (8)	0.0050 (7)
C2	0.0241 (8)	0.0201 (8)	0.0438 (11)	0.0008 (6)	0.0020 (8)	0.0045 (7)
C3	0.0166 (7)	0.0280 (8)	0.0257 (8)	−0.0021 (6)	−0.0004 (6)	0.0010 (7)
C4	0.0189 (7)	0.0217 (7)	0.0239 (8)	−0.0016 (6)	0.0000 (6)	−0.0011 (6)
C5	0.0177 (7)	0.0172 (7)	0.0176 (7)	−0.0041 (5)	0.0004 (6)	0.0026 (6)
C6	0.0186 (7)	0.0210 (8)	0.0349 (9)	−0.0013 (6)	−0.0035 (7)	−0.0042 (7)
C7	0.0230 (8)	0.0215 (8)	0.0328 (9)	−0.0044 (6)	0.0002 (7)	−0.0052 (7)
C8	0.0173 (7)	0.0163 (7)	0.0168 (7)	−0.0037 (5)	0.0011 (5)	0.0019 (5)
C9	0.0162 (7)	0.0188 (7)	0.0198 (7)	−0.0004 (5)	0.0022 (6)	0.0009 (6)
C10	0.0203 (7)	0.0171 (7)	0.0191 (7)	−0.0006 (5)	0.0030 (6)	−0.0018 (6)
C12	0.0222 (7)	0.0166 (7)	0.0241 (8)	0.0009 (6)	−0.0023 (6)	−0.0016 (6)
C13	0.0192 (7)	0.0213 (7)	0.0246 (8)	0.0022 (6)	−0.0034 (6)	−0.0026 (6)

### Geometric parameters (Å, °)

**Table:**

Tb1—O1^i^	2.3596 (11)	N3—C3	1.346 (2)
Tb1—O1^ii^	2.3596 (11)	N3—C7	1.350 (2)
Tb1—O1	2.3596 (11)	N4—C13	1.351 (2)
Tb1—O1^iii^	2.3596 (11)	N4—C10	1.3533 (19)
Tb1—O3^i^	2.4097 (10)	C3—C4	1.378 (2)
Tb1—O3^ii^	2.4097 (10)	C3—H3	0.9300
Tb1—O3	2.4097 (10)	C4—C5	1.400 (2)
Tb1—O3^iii^	2.4097 (10)	C4—H4	0.9300
Mo1—C1^iv^	2.1715 (18)	C5—C6	1.400 (2)
Mo1—C1	2.1715 (18)	C5—C8	1.480 (2)
Mo1—C1^v^	2.1715 (18)	C6—C7	1.379 (2)
Mo1—C1^vi^	2.1715 (18)	C6—H6	0.9300
Mo1—C2^iv^	2.1731 (18)	C7—H7	0.9300
Mo1—C2	2.1731 (18)	C8—C12	1.399 (2)
Mo1—C2^vi^	2.1731 (18)	C8—C9	1.403 (2)
Mo1—C2^v^	2.1731 (18)	C9—C10	1.380 (2)
O1—N3	1.3338 (17)	C9—H9	0.9300
O2—N4	1.3323 (16)	C10—H10	0.9300
O3—H3WA	0.8326	C12—C13	1.377 (2)
O3—H3WB	0.8422	C12—H12	0.9300
N1—C1	1.155 (2)	C13—H13	0.9300
N2—C2	1.152 (2)		
			
O1^i^—Tb1—O1^ii^	96.562 (17)	C1^iv^—Mo1—C2^v^	143.33 (6)
O1^i^—Tb1—O1	96.562 (17)	C1—Mo1—C2^v^	76.77 (7)
O1^ii^—Tb1—O1	140.48 (5)	C1^v^—Mo1—C2^v^	74.88 (7)
O1^i^—Tb1—O1^iii^	140.48 (5)	C1^vi^—Mo1—C2^v^	140.36 (6)
O1^ii^—Tb1—O1^iii^	96.562 (17)	C2^iv^—Mo1—C2^v^	116.87 (11)
O1—Tb1—O1^iii^	96.562 (17)	C2—Mo1—C2^v^	74.10 (5)
O1^i^—Tb1—O3^i^	75.68 (4)	C2^vi^—Mo1—C2^v^	74.10 (5)
O1^ii^—Tb1—O3^i^	146.10 (4)	N3—O1—Tb1	126.90 (9)
O1—Tb1—O3^i^	73.39 (4)	Tb1—O3—H3WA	128.0
O1^iii^—Tb1—O3^i^	72.73 (4)	Tb1—O3—H3WB	120.9
O1^i^—Tb1—O3^ii^	73.39 (4)	H3WA—O3—H3WB	100.0
O1^ii^—Tb1—O3^ii^	75.68 (4)	O1—N3—C3	120.24 (14)
O1—Tb1—O3^ii^	72.73 (4)	O1—N3—C7	119.45 (14)
O1^iii^—Tb1—O3^ii^	146.10 (4)	C3—N3—C7	120.30 (14)
O3^i^—Tb1—O3^ii^	130.38 (3)	O2—N4—C13	118.60 (13)
O1^i^—Tb1—O3	146.10 (4)	O2—N4—C10	120.38 (13)
O1^ii^—Tb1—O3	72.73 (4)	C13—N4—C10	121.00 (13)
O1—Tb1—O3	75.68 (4)	N1—C1—Mo1	175.79 (19)
O1^iii^—Tb1—O3	73.39 (4)	N2—C2—Mo1	176.07 (18)
O3^i^—Tb1—O3	130.38 (3)	N3—C3—C4	121.02 (15)
O3^ii^—Tb1—O3	72.79 (5)	N3—C3—H3	119.5
O1^i^—Tb1—O3^iii^	72.73 (4)	C4—C3—H3	119.5
O1^ii^—Tb1—O3^iii^	73.39 (4)	C3—C4—C5	120.65 (15)
O1—Tb1—O3^iii^	146.10 (4)	C3—C4—H4	119.7
O1^iii^—Tb1—O3^iii^	75.68 (4)	C5—C4—H4	119.7
O3^i^—Tb1—O3^iii^	72.79 (5)	C6—C5—C4	116.49 (14)
O3^ii^—Tb1—O3^iii^	130.38 (3)	C6—C5—C8	121.17 (14)
O3—Tb1—O3^iii^	130.38 (3)	C4—C5—C8	122.23 (14)
C1^iv^—Mo1—C1	74.50 (5)	C7—C6—C5	121.10 (15)
C1^iv^—Mo1—C1^v^	117.74 (11)	C7—C6—H6	119.4
C1—Mo1—C1^v^	74.50 (5)	C5—C6—H6	119.4
C1^iv^—Mo1—C1^vi^	74.50 (5)	N3—C7—C6	120.42 (16)
C1—Mo1—C1^vi^	117.74 (11)	N3—C7—H7	119.8
C1^v^—Mo1—C1^vi^	74.50 (5)	C6—C7—H7	119.8
C1^iv^—Mo1—C2^iv^	74.88 (7)	C12—C8—C9	116.87 (14)
C1—Mo1—C2^iv^	140.36 (6)	C12—C8—C5	120.77 (13)
C1^v^—Mo1—C2^iv^	143.33 (6)	C9—C8—C5	122.32 (13)
C1^vi^—Mo1—C2^iv^	76.77 (7)	C10—C9—C8	120.66 (14)
C1^iv^—Mo1—C2	76.77 (7)	C10—C9—H9	119.7
C1—Mo1—C2	74.88 (7)	C8—C9—H9	119.7
C1^v^—Mo1—C2	140.36 (6)	N4—C10—C9	120.16 (14)
C1^vi^—Mo1—C2	143.33 (6)	N4—C10—H10	119.9
C2^iv^—Mo1—C2	74.10 (5)	C9—C10—H10	119.9
C1^iv^—Mo1—C2^vi^	140.36 (6)	C13—C12—C8	120.98 (14)
C1—Mo1—C2^vi^	143.33 (6)	C13—C12—H12	119.5
C1^v^—Mo1—C2^vi^	76.77 (7)	C8—C12—H12	119.5
C1^vi^—Mo1—C2^vi^	74.88 (7)	N4—C13—C12	120.17 (14)
C2^iv^—Mo1—C2^vi^	74.10 (5)	N4—C13—H13	119.9
C2—Mo1—C2^vi^	116.87 (11)	C12—C13—H13	119.9

Symmetry codes: (i) −*y*+1, *x*−1/2, −*z*; (ii) −*x*+3/2, −*y*+1/2, *z*; (iii) *y*+1/2, −*x*+1, −*z*; (iv) −*y*+3/2, *x*, *z*; (v) *y*, −*x*+3/2, *z*; (vi) −*x*+3/2, −*y*+3/2, *z*.

### Hydrogen-bond geometry (Å, °)

**Table:**

*D*—H···*A*	*D*—H	H···*A*	*D*···*A*	*D*—H···*A*
O3—H3WA···O2^vii^	0.83	1.85	2.6702 (15)	169
O3—H3WB···O2^viii^	0.84	1.92	2.7417 (16)	164
O3—H3WB···N4^viii^	0.84	2.62	3.4251 (16)	161

Symmetry codes: (vii) −*y*+1/2, *x*, *z*+1; (viii) *x*+1/2, *y*+1/2, −*z*.
